# Urine Donor–Derived Cell-Free DNA Helps Discriminate BK Polyomavirus-Associated Nephropathy in Kidney Transplant Recipients With BK Polyomavirus Infection

**DOI:** 10.3389/fimmu.2020.01763

**Published:** 2020-08-19

**Authors:** Xu-Tao Chen, Wen-Fang Chen, Jun Li, Rong-Hai Deng, Yang Huang, Shi-Cong Yang, Pei-Song Chen, Ting-Ya Jiang, Hai-Tao Liu, Chang-Xi Wang, Li-Zhong Chen, Jiang Qiu, Gang Huang

**Affiliations:** ^1^Department of Organ Transplant, The First Affiliated Hospital of Sun Yat-sen University, Guangzhou, China; ^2^Guangdong Provincial Key Laboratory of Organ Donation and Transplant Immunology, The First Affiliated Hospital, Sun Yat-sen University, Guangzhou, China; ^3^Guangdong Provincial International Cooperation Base of Science and Technology (Organ Transplantation), The First Affiliated Hospital, Sun Yat-sen University, Guangzhou, China; ^4^Department of Pathology, The First Affiliated Hospital of Sun Yat-sen University, Guangzhou, China; ^5^Department of Clinical Laboratory, The First Affiliated Hospital of Sun Yat-sen University, Guangzhou, China; ^6^AlloDx Biotech. Co., Suzhou Industrial Park, Suzhou, China

**Keywords:** kidney transplantation, BK polyomavirus, BK polyomavirus-associated nephropathy, donor-derived cell-free DNA, area under the curve, prediction

## Abstract

**Background:** Studies have shown that plasma donor–derived cell-free DNA (dd-cfDNA) can predict renal allograft antibody-mediated rejection. This study was performed to evaluate the value of urine dd-cfDNA concentration and dd-cfDNA fraction (%) for discriminating BK polyomavirus-associated nephropathy (BKPyVAN) in kidney transplant recipients with urinary BK polyomavirus (BKPyV) infection.

**Methods:** In this retrospective single-center observational study, we enrolled kidney transplant recipients who were diagnosed with urine BKPyV infection between August 2018 and May 2019 at the First Affiliated Hospital of Sun Yat-sen University. Urine dd-cfDNA was measured by using a novel target region capture sequencing methodology. The pathological diagnosis of BKPyVAN was confirmed by anti-SV40-T immunohistochemical staining and classified using the American Society for Transplantation schema. Receiver operating characteristic curve analysis was used to investigate the relations of urine dd-cfDNA and dd-cfDNA% to intrarenal allograft BKPyV infection states.

**Results:** In total, 93 patients were enrolled, including 40 cases of proven BKPyVAN, seven cases of probable BKPyVAN, 23 cases of possible BKPyVAN, and 23 cases of resolving BKPyVAN. Urine dd-cfDNA level in proven BKPyVAN (22.09 ± 21.27 ng/ml) was comparable to that in probable BKPyVAN (15.64 ± 6.73 ng/ml, *P* = 0.434) but was significantly higher than that in possible BKPyVAN (5.60 ± 3.53 ng/ml) and resolving BKPyVAN (5.30 ± 3.34 ng/ml) (both *P*s < 0.05). Urine dd-cfDNA% of proven BKPyVAN (0.71 ± 0.21) was lower than that of probable BKPyVAN (0.91 ± 0.04, *P* < 0.001), but was significantly higher than that of possible BKPyVAN (0.56 ± 0.30) and resolving BKPyVAN (0.46 ± 0.28) (both *P*s < 0.05). For distinguishing biopsy-proven BKPyVAN from biopsy-excluded BKPyVAN, the discrimination capacity of urine dd-cfDNA (AUC: 0.842, 95% CI: 0.735, 0.918) was superior to that of plasma BKPyV DNA load (AUC: 0.660, 95% CI: 0.537, 0.769) with 0.181 (95% CI: 0.043, 0.319) difference between areas under ROC curves (*P* = 0.010).

**Conclusion:** The elevated urine dd-cfDNA level may help discriminate BKPyVAN in kidney transplant recipients with BKPyV viruria.

## Introduction

BK polyomavirus-associated nephropathy (BKPyVAN) can cause renal allograft injury and loss (1). The primary methods for detecting BK polyomavirus (BKPyV) infection are urine cytological examination and quantitative polymerase chain reaction (PCR) for detecting BKPyV DNA in urine and plasma. The gold standard for diagnosing BKPyVAN is a kidney biopsy, which is associated with potential complications, sampling errors, and poor reproducibility (2).

One proposed method for detecting organ injury is the measurement of cell-free DNA (cfDNA) in body fluids (3). Studies have demonstrated that kidney (4, 5), liver (6), heart (7), and lung (8) transplant recipients with acute rejection have increased plasma dd-cfDNA as compared with recipients with stable graft function. As such, donor-derived cell-free DNA (dd-cfDNA) may serve as a non-invasive biomarker of allograft injury/rejection.

As with acute antibody-mediated rejection, renal allograft injury due to BKPyV infection may also result in an elevated level of dd-cfDNA in the urine or blood. Bloom et al. reports that the percentage of plasma dd-cfDNA (dd-cfDNA%) in two patients with BKPyVAN was 4.6 and 2.3%, respectively, which were higher than the 1.0% cutoff of discriminating antibody-mediated rejection (4). Whitlam et al. reports the plasma dd-cfDNA% was 1.5% in one patient with BKPyVAN (9). However, another study finds that plasma dd-cfDNA% remained below the threshold value (0.88%) in three cases of BKPyVAN (10). The majority of research on the use of dd-cfDNA for diagnosing acute rejection/injury has been focused on the proportion of dd-cfDNA in plasma. When graft microvascular endothelial cells are damaged, a cardinal feature of acute rejection, dd-cfDNA is released into the blood thereby resulting in an increase in plasma dd-cfDNA (11). However, the BKPyV mainly infects and causes damage in renal tubular epithelial cells rather than vascular endothelial cells (12). We may, therefore, speculate that dd-cfDNA fragments from damaged tubular epithelial cells due to BKPyV infection are released into the urine, causing an increase in ratio and absolute quantification of urine dd-cfDNA.

Currently, the relationship between urine dd-cfDNA and BKPyVAN has not been confirmed. We performed this study to evaluate the relationship between urine dd-cfDNA and intrarenal allograft BKPyV infection states in adult kidney transplant recipients.

## Materials and Methods

### Study Population and Samples

In this retrospective single-center observational study, all kidney transplant recipients who were diagnosed with urine BKPyV infection at our hospital between August 2018 and May 2019 were assessed for eligibility for inclusion. The exclusion criteria were (1) urinary tract bacterial infection; (2) age < 18 years; (3) pediatric donor or pediatric recipient; (4) multiorgan, en bloc, or repeated kidney transplant; (5) positive antidonor-specific antibody; and (6) concurrent T cell-mediated rejection (manifested as Banff endarteritis ≥ 1). This study adhered to the tenets of the Declaration of Helsinki and was approved by the Ethics Committee of the First Affiliated Hospital of Sun Yat-sen University (No. [2019] 221). Written informed consent was obtained from all patients. Kidney allografts from living or deceased organ donors who met the ethical guidelines for kidney donation were used.

First, all enrolled patients were grouped according to the 2019 American Society of Transplantation guidelines (13), including (1) possible BKPyVAN defined by urine BKPyV DNA load > 7 log_10_ copies/mL with negative BK viremia and negative anti-SV40-T immunohistochemical (IHC) staining on kidney biopsy, (2) probable BKPyVAN defined by sustained plasma BKPyV DNA load >3 log_10_ copies/mL in two measurements within 3 weeks with negative anti-SV40-T IHC staining on kidney biopsy, (3) presumptive BKPyVAN defined by plasma BKPyV DNA load >4 log_10_ copies/mL in at least one of two measurements in <3 weeks with negative anti-SV40-T IHC staining on kidney biopsy, and (4) proven BKPyVAN defined by urine BKPyV DNA load >7 log_10_ copies/mL with positive anti-SV40-T IHC staining on kidney biopsy. Second, to evaluate the level of urine dd-cfDNA during BKPyVAN remission, patients with resolving BKPyVAN (who had been previously diagnosed with biopsy-proved BKPyVAN) were included simultaneously. The criteria used to define the resolving BKPyVAN was described elsewhere (14), including (1) BK viremia becoming negative, (2) urine BKPyV DNA load decreasing by >2 log_10_ copies/mL, (3) serum creatinine remaining stable or decreasing without anti-rejection therapy, and (4) anti-SV40-T IHC staining becoming negative without signs of rejection on repeated kidney biopsy. Table 1 describes the clinicopathological progress of patients with resolving BKPyVAN. In this group of patients, serum creatinine remained stable, plasma and urine BKPyV DNA load decreased significantly, and anti-SV40-T IHC staining on kidney biopsies turned negative.

**Table 1:** Clinical follow-up data of the resolving BKPyVAN group.

	**At diagnosis of proved BKPyVAN (*N* = 23)**	**At diagnosis of resolving BKPyVAN (*N* = 23)**	***P***
Interval time (months)	–	20.3 (IQR: 17.5, 36.1)	–
Serum creatinine (μmol/L)	171.0 (IQR: 142.0, 187.0)	151.0 (IQR: 140.0, 203.0)	0.703
Urine viral load (copies/mL)	9.8 × 10^8^ (IQR: 2.2 × 10^8^, 3.9 × 10^10^)	2.9 × 10^7^ (IQR: 1.1 × 10^7^, 1.1 × 10^8^)	<0.001
Plasma viral load (copies/mL)	2.8 × 10^3^ (IQR: 0, 1.8 × 10^5^)	0 (IQR: 0, 0)	<0.001
Extent of anti-SV40-T IHC staining	11.0% (IQR: 6.0%, 16.3%)	0 (IQR: 0, 0)	<0.001

*BKPyVAN, BK polyomavirus-associated nephropathy; IQR, interquartile range; IHC, immunohistochemical*.

### Urine cfDNA Isolation and Blood Genomic DNA Extraction

Urine and blood samples were harvested within 48 h of kidney biopsy. All patients were fasted for more than 8 h before collecting samples. No patient experienced oliguria or anuria in this study. A total of 8 ml of midmorning urine as well as 8 ml of blood were collected in cfDNA Collecting Tubes (Streck, Cat. No. 218962, NE, USA). Diazolidinyl urea (final concentration 3%) and SUPERase•In™ RNase Inhibitor (final concentration 5%) were added into the tubes to prevent DNA degradation. Specimens were stored at 4°C and airlifted to the laboratory. The samples were centrifuged at 16,000 × g for 10 min. Three ml of the urine supernatant was collected, and the total cfDNA was extracted using the Circulating Nucleic Acid Kit (Qiagen, Cat. No. 55114, Germany). The extraction range of the kit is 1 ng/mL−1 ug/mL, and this linear range can ensure a stable extraction volume between samples. The white blood cells were separated in the blood sample, and the total germline genomic DNA (gDNA) was extracted using the QIAamp DNA Blood Mini Kit (Qiagen, Cat. No. 51104, Germany). A total of 5.6 μL carrier RNA was added in every 4 mL of urine. All samples were processed according to the kit manufacturer's instructions.

### Library Construction and Target Region Capture Sequencing

The purified cfDNA and gDNA were quantified using a Qubit fluorometer (Life Technologies, Cat. No. Q33216, China). Quality control of cfDNA and gDNA was based on spectrophotometric analysis at a 260–280 nm ratio.

Next, 30 ng of total cfDNA out of 30 ng of gDNA was used for DNA library construction with a KAPA LTP library preparation kit (KAPA, Cat. No. KK8235, USA). The target region included 6,200 human single-nucleotide polymorphism (SNP) loci with a custom TruGrade® DNA Oligos pool (IDT, California, USA) according to the manufacturer's protocol, and SNPs were selected for their high minor allele frequency in the population (MAF = 0.4–0.6). Captured libraries were characterized using an Agilent 2100 Bioanalyzer (Agilent, Cat. No. 2200, USA), then pooled and sequenced (Illumina X-ten, 10 ± 5 million; paired end [PE] 150 bp).

### Bioinformatics and dd-cfDNA Quantification

Sequencing raw data were trimmed by removing low-quality reads, adapter contamination reads, and PCR duplications. Then, reads were aligned against the Genome Reference Consortium Human Build 38 (www.ncbi.nlm.nih.gov/assembly/GCF_000001405.38) using a Burrows-Wheeler Aligner (www.bio-bwa.sourceforge.net). Samtools (http://samtools.sourceforge.net/) were used to conduct SNP calling.

The read numbers were counted for each allele of the 6200 SNPs. The minor allele ratio values for the informative SNP locus (recipients' homozygous SNP loci with at least one alternative allele read) were calculated for dd-cfDNA quantification. A Bayes approach was utilized to quantify the dd-cfDNA level. For each informative SNP, a binomial model was used to estimate the donor-derived allele frequency, and the donor genotype was estimated based on the donor-specific allele frequency in the population (15). The amount of dd-cfDNA (nanogram, ng) was normalized against urine volume. Absolute quantification of dd-cfDNA per mL urine is calculated by multiplying the total concentration of cfDNA in a sample by the dd-cfDNA fraction (%).

$$\begin{array}{l} ddcfDNA_{urine}=\frac{C_{cfDNA}\times V_{elution}\times ddcfDNA\left(\%\right)}{V_{urine}} \end{array}$$

*Dd-cfDNA*_*urine*_ is the absolute concentration of target within the urine per mL (ng/mL), *C*_*cfDNA*_ is the extraction concentration of total cfDNA (ng/uL), *V*_*elution*_ is the volume of eluent used during the cfDNA extraction process in μL, and *V*_*urine*_ is the volume of urine used for cfDNA extraction in mL; dd-cfDNA (%) is the ratio of dd-cfDNA to total cfDNA.

### Virological Studies

BKPyV viral load in urine and plasma samples were determined by a quantitative PCR Detection Kit (Sinomed, Beijing, China) as described elsewhere (16). Assays were performed on an ABI Prism 7500 Sequence Detection system (Applied Biosystems, USA), according to the manufacturer's instructions. PCR amplifications were performed in a 25-μL reaction volume that contained 5 μL of extracted DNA. Probes were designed to specifically detect the BKPyV and did not cross-react with sequences present in the related JC or SV-40 polyomavirus as per the manufacturer's statement. The lower limit of quantitation was 1 × 10^3^ copies/mL for both plasma and urine.

### Diagnosis of BKPyVAN

Pathological lesions were scored according to the 2017 Banff criteria (17). The pathological diagnosis of proven BKPyVAN was confirmed by immunohistochemical (IHC) staining as previously described (18). The histological features of BKPyVAN were classified using the American Society for Transplantation schema, and BKPyVAN was classified as stage A, B, or C based on the guidelines published by Hirsch et al. (19).

### Statistical Analysis

Continuous data were reported as mean ± standard deviation, and categorical data as number (%). Both parametric and non-parametric inferential statistics were used in this study, depending on the assumption of data normality. For comparisons of all four groups, according to each outcome's normality assumptions, one-way ANOVA (normality assumed) and Kruskal-Wallis test (non-parametric, non-normality) tests were used, and Fisher's LSD comparison was used for *post hoc* analysis. Repeated measurement of urine dd-cfDNA was compared by the paired-sample *T*-test.

Receiver operating characteristic (ROC) curve analysis was used to investigate the relations of independent variables to the intrarenal BKPyV infection state. Discrimination capacity indexes were reported, including the area under the ROC curve (AUC), sensitivity, specificity, positive predictive value, negative predictive value, positive likelihood ratio, negative likelihood ratio, diagnostic odds ratio, and Youden index. Correlation coefficient analyses were performed to determine the correlation coefficients between individual pathological scores or serum creatinine and urine dd-cfDNA and dd-cfDNA%. Spearman's correlation analysis was used for variables with ordinal/rank properties, and Pearson's correlation analysis was used for variables with interval/continuous properties. All statistical analyses were two-tailed, and values of *P* < 0.05 were considered to indicate statistical significance. All analyses were performed using IBM SPSS version 20 software (IBM Corporation, Somers, New York).

## Results

### Patients

**Figure 1:**
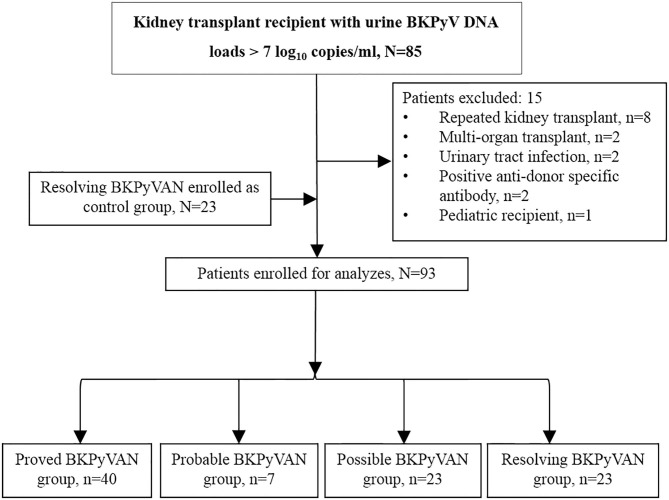
Patient inclusion flow diagram. BKPyVAN, BK polyomavirus-associated nephropathy; BKPyV, BK polyomavirus; TCMR, T cell-mediated rejection.

A flow diagram of patient inclusion is shown in Figure 1. Ninety-three patients were enrolled, including 54 (58.06%) males and 39 (41.94%) females with a mean age of 40.31 ± 10.40 years (median: 38.79 years, interquartile range [IQR]: 34.25–46.62 years). Patients were allocated to four groups according to their BKPyV infection state, and there were 40 cases of proved BKPyVAN, seven cases of probable BKPyVAN, 23 cases of possible BKPyVAN, and 23 cases of resolving BKPyVAN. No patient was diagnosed with presumptive BKPyVAN during the study period. No patient in the resolving BKPyVAN group was from the biopsy-proven BKPyVAN group. Patient characteristics are presented and compared in Table 2. The serum creatinine in the proved BKPyVAN group (210.63 ± 89.82 μmol/L) was greater than that in the possible BKPyVAN group (114.57 ± 26.73 μmol/L, *P* < 0.001) but was comparable to that in the probable BKPyVAN group (153.86 ± 51.67 μmol/L, *P* = 0.113) and in the resolving BKPyVAN group (174.30 ± 54.04 μmol/L, *P* = 0.083). The serum creatinine in the probable BKPyVAN group was slightly higher than that in the possible BKPyVAN group (153.86 ± 51.67 μmol/L vs. 114.57 ± 26.73 μmol/L), but the difference was not significant (*P* = 0.184). The urine BKPyV viral load in the proved BKPyVAN group was similar to that in the probable BKPyVAN group, but significantly higher than that in the possible BKPyVAN group (*P* = 0.007) and resolving BKPyVAN group (*P* = 0.005). Plasma BKPyV viral load was similar between the proved BKPyVAN group and the probable BKPyVAN group (*P* = 0.317). No patient in the possible BKPyVAN group or in the resolving BKPyVAN group had BK viremia.

**Table 2:** Patient characteristics.

**Parameter**	**All patients** **(*N* = 93)**	**Proven BKPyVAN** **(*n* = 40)**	**Probable BKPyVAN** **(*n* = 7)**	**Possible BKPyVAN** **(*n* = 23)**	**Resolving BKPyVAN** **(*n* = 23)**	P^*^
**Demographic characteristics**						
Age at sample collection (years)	40.31 ± 10.40	40.21 ± 10.44	36.23 ± 9.06	39.44 ± 10.78	42.62 ± 10.44	0.503
Sex						0.406
Male	54 (58.06)	20 (50.00)	5 (71.43)	16 (69.57)	13 (56.52)	
Female	39 (41.94)	20 (50.00)	2 (28.57)	7 (30.43)	10 (43.48)	
Height (cm)	164.01 ± 9.34	162.83 ± 10.24	167.86 ± 7.31	166.78 ± 9.25	162.13 ± 7.73	0.188
Weight (kg)	55.22 ± 11.74	53.10 ± 11.69	59.57 ± 11.43	60.28 ± 11.63	52.53 ± 10.71	0.049
Body mass index (kg/m^2^)	20.38 ± 3.21	19.93 ± 3.44	20.97 ± 2.74	21.49 ± 2.88	19.87 ± 3.11	0.231
Indication for renal transplantation						0.705
IgA nephropathy	12 (12.90)	5 (12.50)	0 (0.00)	4 (17.39)	3 (13.04)	
Focal segmental glomerular sclerosis	2 (2.15)	1 (2.50)	0 (0.00)	0 (0.00)	1 (4.35)	
Nephrotic syndrome	3 (3.23)	0 (0.00)	0 (0.00)	2 (8.70)	1 (4.35)	
Chronic glomerulonephritis	22 (23.66)	9 (22.50)	2 (28.57)	6 (26.09)	5 (21.74)	
Lupus nephritis	3 (3.23)	1 (2.50)	0 (0.00)	0 (0.00)	2 (8.70)	
Diabetic nephropathy	4 (4.30)	2 (5.00)	1 (14.29)	1 (4.35)	0 (0.00)	
Hypertensive nephropathy	1 (1.08)	1 (2.50)	0 (0.00)	0 (0.00)	0 (0.00)	
Others/unknown	46 (49.46)	21 (52.50)	4 (57.14)	10 (43.48)	11 (47.83)	
**Transplant characteristics**						
Donor type						0.685
Deceased	82 (88.17)	37 (92.50)	6 (85.71)	20 (86.96)	19 (82.61)	
Living	11 (11.83)	3 (7.50)	1 (14.29)	3 (13.04)	4 (17.39)	
Induction agent						0.275
Basiliximab	23 (24.73)	9 (22.50)	1 (14.29)	4 (17.39)	9 (39.13)	
Thymoglobulin	68 (73.12)	30 (75.00)	5 (71.43)	19 (82.61)	14 (60.87)	
Basiliximab + thymoglobulin	2 (2.15)	1 (2.50)	1 (14.29)	0 (0.00)	0 (0.00)	
Delayed graft function						0.631
No	84 (90.32)	36 (90.00)	7 (100.00)	20 (86.96)	21 (91.30)	
Yes	9 (9.68)	4 (10.00)	0 (0.00)	3 (13.04)	2 (8.70)	
Renal replacement therapy: hemodialysis						0.665
No	36 (38.71)	14 (35.00)	4 (57.14)	8 (34.78)	10 (43.48)	
Yes	57 (61.29)	26 (65.00)	3 (42.86)	15 (65.22)	13 (56.52)	
Renal replacement therapy: peritoneal dialysis						0.669
No	66 (70.97)	27 (67.50)	4 (57.14)	18 (78.26)	17 (73.91)	
Yes	27 (29.03)	13 (32.50)	3 (42.86)	5 (21.74)	6 (26.09)	
Length of renal replacement therapy (months)	20.45 ± 26.60	18.04 ± 22.97	35 ± 43.87	24.62 ± 30.81	16.04 ± 20.79	0.309
Length of time post-transplant (months)	21.84 ± 23.05	17.43 ± 15.33	8.24 ± 9.29	7.55 ± 5.08	47.94 ± 27.14	<0.001
**Clinical characteristics**						
Baseline serum creatinine (μmol/L)	114.76 ± 34.15	112.78 ± 37.53	111.57 ± 23.06	113.48 ± 34.19	120.48 ± 31.94	0.837
Serum creatinine at sample collection (μmol/L)	173.61 ± 77.21	210.63 ± 89.82	153.86 ± 51.67	114.57 ± 26.73	174.30 ± 54.04	<0.001
Maintenance immunosuppression						
Mycophenolate	93 (100.00)	40 (100.00)	7 (100.00)	23 (100.00)	23 (100.00)	1.000
Tacrolimus	92 (98.92)	40 (100.00)	7 (100.00)	23 (100.00)	22 (95.65)	0.419
Cyclosporin	1 (1.08)	0 (0.00)	0 (0.00)	0 (0.00)	1 (4.35)	0.419
Corticosteroids	93 (100.00)	40 (100.00)	7 (100.00)	23 (100.00)	23 (100.00)	1.000
Urine viral load (copies/mL)	8.2 × 10^7^	7.1 × 10^8^	5.4 × 10^8^	4.1 × 10^7^	2.9 × 10^7^	<0.001
	(IQR: 2.4 × 10^7^, 1.5 × 10^9^)	(IQR: 3.2 × 10^7^, 5.3 × 10^9^)	(IQR: 2.5 × 10^8^, 1.1 × 10^10^)	(IQR: 1.8 × 10^7^, 2.1 × 10^8^)	(IQR: 1.1 × 10^7^, 1.1 × 10^8^)	
Plasma viral load (copies/mL)	0 (0, 1.9 × 10^3^)	1.4 × 10^3^ (0, 3.5 × 10^4^)	1.7 × 10^3^ (1.0 × 10^3^, 8.3 × 10^3^)	0 (0, 0)	0 (0, 0)	<0.001

*BKPyVAN, BK polyomavirus-associated nephropathy; BKPyV, BK polyomavirus; IQR, interquartile range*.

* *Comparison among Proven BKPyVAN, Probable BKPyVAN, Possible BKPyVAN, and Resolving BKPyVAN*.

### Dd-cfDNA and dd-cfDNA% in Urine

The urine total cfDNA in the proved BKPyVAN group (35.27 ± 31.74 ng/ml) was significantly higher than that in the possible BKPyVAN group (12.32 ± 9.15 ng/ml; *P* < 0.001) and the resolving BKPyVAN group (16.36 ± 16.34 ng/ml; *P* = 0.002) but was slightly higher than that in the probable BKPyVAN group (17.16 ± 7.00 ng/ml; *P* = 0.058). The urine total cfDNA in the probable BKPyVAN group (17.16 ± 7.00 ng/ml) was comparable to that in the possible BKPyVAN group (12.32 ± 9.15 ng/ml; *P* = 0.629) and the resolving BKPyVAN group (16.36 ± 16.34 ng/ml; *P* = 0.936).

**Figure 2:**
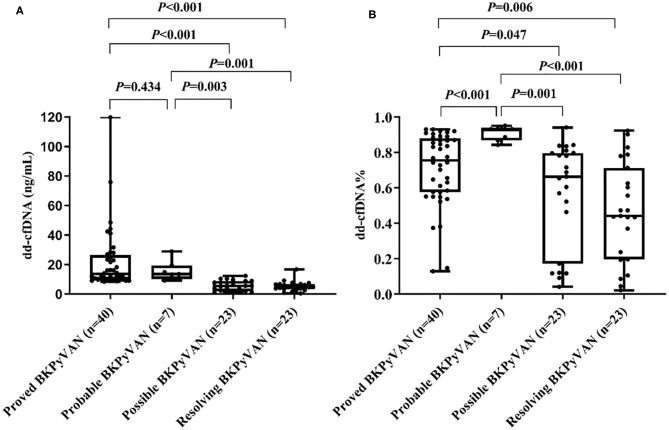
Box plots of **(A)** urine dd-cfDNA and **(B)** urine dd-cfDNA% of the four groups.

The urine dd-cfDNA in the proved BKPyVAN group (22.09 ± 21.27 ng/ml) was significantly higher than that in the possible BKPyVAN group (5.60 ± 3.53 ng/ml; *P* < 0.001) and the resolving BKPyVAN group (5.30 ± 3.34 ng/ml; *P* < 0.001). Similarly, the urine dd-cfDNA in the probable BKPyVAN group (15.64 ± 6.73 ng/ml) was higher than that in the possible BKPyVAN group (5.60 ± 3.53 ng/ml; *P* = 0.003) and the resolving BKPyVAN group (5.30 ± 3.34 ng/ml; *P* = 0.001) (Figure 2A). The difference of urine dd-cfDNA between the proved BKPyVAN group (22.09 ± 21.27 ng/ml) and the probable BKPyVAN group (15.64 ± 6.73 ng/ml) was not significant (*P* = 0.434).

The urine dd-cfDNA% of the proved BKPyVAN group (0.71 ± 0.21) was significantly higher than that of the possible BKPyVAN group (0.56 ± 0.30; *P* = 0.047) and the resolving BKPyVAN group (0.46 ± 0.28; *P* = 0.006). Similarly, the urine dd-cfDNA% in the probable BKPyVAN group (0.91 ± 0.04) was higher than that in the possible BKPyVAN group (0.56 ± 0.30; *P* = 0.001) and the resolving BKPyVAN group (0.46 ± 0.28; *P* < 0.001) (Figure 2B). The urine dd-cfDNA% of the proved BKPyVAN group (0.71 ± 0.21) was lower than that of the probable BKPyVAN group (0.91 ± 0.04, *P* < 0.001). No significant correlation was found between plasma BKPyV load and urine dd-cfDNA (*P* = 0.322) or urine dd-cfDNA% (*P* = 0.424).

### Discrimination Capacity of dd-cfDNA and dd-cfDNA%

**Figure 3:**
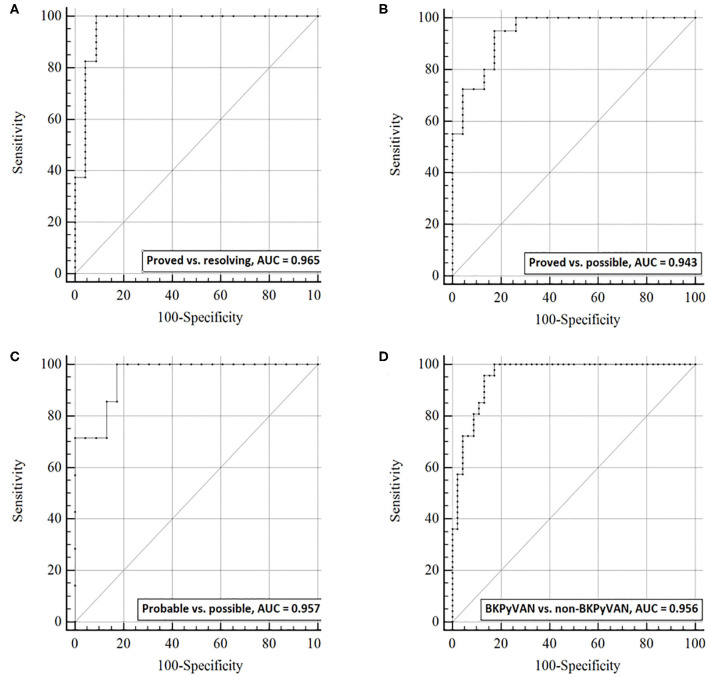
The discrimination capacity of urine dd-cfDNA. Receiver operating characteristic curves of dd-cfDNA for **(A)** distinguishing proven BKPyVAN from resolving BKPyVAN; **(B)** distinguishing proven BKPyVAN from possible BKPyVAN; **(C)** distinguishing probable BKPyVAN from possible BKPyVAN; **(D)** distinguishing BKPyVAN (proven BKPyVAN + probable BKPyVAN) from non-BKPyVAN (possible BKPyVAN + resolving BKPyVAN).

**Figure 4:**
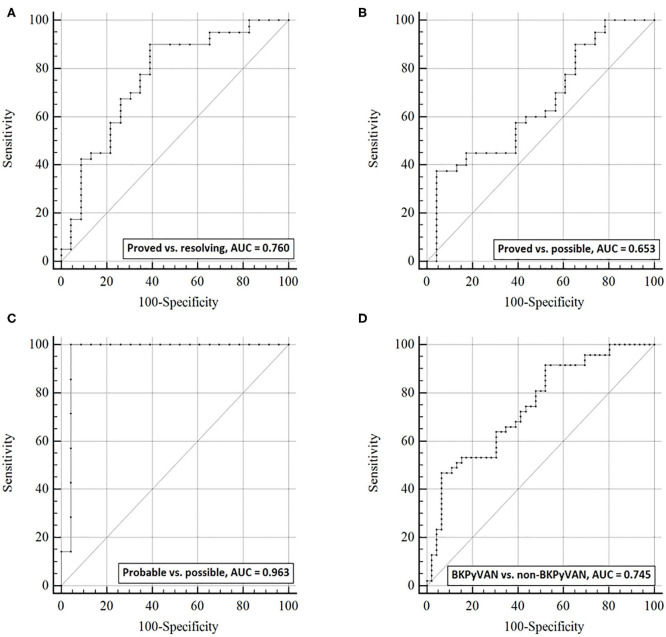
The discrimination capacity of urine dd-cfDNA%. Receiver operating characteristic curves of dd-cfDNA% for **(A)** distinguishing proven BKPyVAN from resolving BKPyVAN; **(B)** distinguishing proven BKPyVAN from possible BKPyVAN; **(C)** distinguishing probable BKPyVAN from possible BKPyVAN; **(D)** distinguishing BKPyVAN (proven BKPyVAN + probable BKPyVAN) from non-BKPyVAN (possible BKPyVAN + resolving BKPyVAN).

ROC curves analysis was used to examine the discrimination capacity of urine dd-cfDNA (Figure 3) and dd-cfDNA% (Figure 4) between the four defined groups. The AUC of urine dd-cfDNA for distinguishing proven BKPyVAN vs. resolving BKPyVAN was 0.965 (95% CI: 0.910, 1.000, Figure 3A), for proven BKPyVAN vs. possible BKPyVAN was 0.943 (95% CI: 0.888, 0.999, Figure 3B), for probable BKPyVAN vs. possible BKPyVAN was 0.957 (95% CI: 0.886, 1.000, Figure 3C), and for BKPyVAN (proven BKPyVAN + probable BKPyVAN) vs. non-BKPyVAN (possible BKPyVAN + resolving BKPyVAN) was 0.956 (95% CI: 0.916, 0.995, Figure 3D).

The AUC of urine dd-cfDNA% for distinguishing proved BKPyVAN vs. resolving BKPyVAN was 0.760 (95% CI: 0.631, 0.889, Figure 4A), for proved BKPyVAN vs. possible BKPyVAN was 0.653 (95% CI: 0.512, 0.794, Figure 4B), for probable BKPyVAN vs. possible BKPyVAN was 0.963 (95% CI: 0.890, 1.000, Figure 4C), and for BKPyVAN (proven BKPyVAN + probable BKPyVAN) vs. non-BKPyVAN (possible BKPyVAN + resolving BKPyVAN) was 0.745 (95% CI: 0.645, 0.844, Figure 4D). The AUC, sensitivity, specificity, positive predictive value, negative predictive value, positive likelihood ratio, negative likelihood ratio, diagnostic odds ratio, and Youden index are presented in Table 3.

**Table 3:** Discrimination capacity of dd-cfDNA and dd-cfDNA% for the diagnosis of BKPyVAN.

	**Discrimination of proven BKPyVAN from resolving BKPyVAN**		**Discrimination of proven BKPyVAN from possible BKPyVAN**		**Discrimination of probable BKPyVAN from possible BKPyVAN**		Discrimination of BKPyVAN^#^ from non-BKPyVAN^*^	
	**dd-cfDNA >** **8.06 ng/ml**	**dd-cfDNA% >** **0.50**	**dd-cfDNA >** **8.93 ng/ml**	**dd-cfDNA% >** **0.84**	**dd-cfDNA >** **9.06 ng/ml**	**dd-cfDNA% >** **0.84**	**dd-cfDNA >** **8.93 ng/ml**	**dd-cfDNA% >** **0.84**
AUC	0.965	0.760	0.943	0.653	0.957	0.963	0.956	0.745
Sensitivity	1.00	0.90	0.95	0.38	1.00	1.00	0.96	0.47
Specificity	0.91	0.61	0.83	0.91	0.83	0.91	0.87	0.91
PPV	0.95	0.80	0.90	0.88	0.64	0.78	0.88	0.85
NPV	1.00	0.78	0.90	0.46	1.00	1.00	0.95	0.63
PLR	11.50	2.30	5.46	4.31	5.75	11.50	7.34	5.38
NLR	0.00	0.16	0.06	0.68	0.00	0.00	0.05	0.58
DOR	420.00	14.00	90.25	6.30	33.25	73.50	150.00	9.24
Youden index	0.91	0.51	0.78	0.29	0.83	0.91	0.83	0.38

*BKPyVAN, BK polyomavirus-associated nephropathy; BKPyV, BK polyomavirus; dd-cfDNA, donor-derived cell-free DNA; AUC, area under the receiver operating characteristics curve; PPV, positive predictive value; NPV, negative predictive value; PLR, positive likelihood ratio; NLR, negative likelihood ratio; DOR, diagnostic odds ratio*.

# BKPyVAN = proved BKPyVAN + probable BKPyVAN;

* *non-BKPyVAN = possible BKPyVAN + resolving BKPyVAN*.

**Figure 5:**
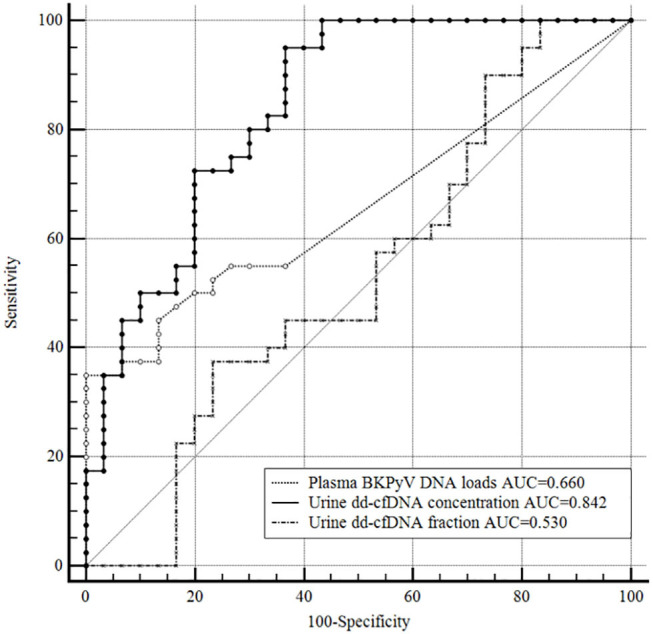
Receiver operating characteristic curves of the plasma BKPyV DNA load, urine dd-cfDNA and urine dd-cfDNA% for the discrimination of biopsy-proved BKPyVAN from biopsy-excluded BKPyVAN (probable BKPyVAN + possible BKPyVAN). The discrimination capacity of urine dd-cfDNA (AUC = 0.842) was superior to that of plasma BKPyV DNA load (AUC = 0.660, *P* = 0.010) and that of urine dd-cfDNA% (AUC = 0.530, *P* < 0.001). The discrimination capacity of urine dd-cfDNA% (AUC = 0.530) was inferior to that of plasma BKPyV DNA load (AUC = 0.660, *P* = 0.147).

An ROC curve analysis was also performed to compare the discrimination capacity of urine dd-cfDNA and plasma BKPyV DNA load for distinguishing biopsy-proven BKPyVAN from biopsy-excluded BKPyVAN (probable BKPyVAN + possible BKPyVAN). As shown in Figure 5, the discrimination capacity of urine dd-cfDNA (AUC: 0.842, 95% CI: 0.735, 0.918) was superior to that of plasma BKPyV DNA load (AUC: 0.660, 95% CI: 0.537, 0.769) with 0.181 (95% CI: 0.043, 0.319) difference between areas under ROC curves (*P* = 0.010). However, the discrimination capacity of urine dd-cfDNA% (AUC: 0.530, 95% CI: 0.407, 0.650) was comparable to that of plasma BKPyV DNA load with 0.130 (95% CI: 0.045, 0.307) difference between areas under ROC curves (*P* = 0.147).

### Serum Creatinine and dd-cfDNA

**Figure 6:**
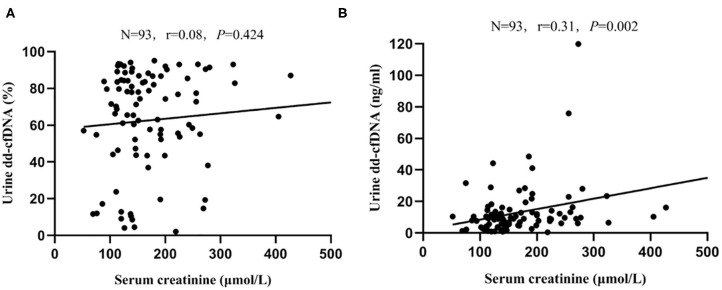
Scatterplot with regression line between serum creatinine level at data collection and **(A)** dd-cfDNA% and **(B)** dd-cfDNA. The correlation coefficient was 0.08 between serum creatinine and dd-cfDNA% (*P* = 0.424), and 0.31 between serum creatinine and dd-cfDNA (*P* = 0.002).

There was no correlation between serum creatinine and dd-cfDNA% (*r* = 0.08, *P* = 0.424, Figure 6A); however, a significant but weak correlation between serum creatinine and dd-cfDNA was observed (*r* = 0.31, *P* = 0.002, Figure 6B). In the BKPyVAN group (proven BKPyVAN + probable BKPyVAN), the level of dd-cfDNA in patients with elevated serum creatinine (>30% increased from baseline serum creatinine) (*n* = 32) was similar to that in patients with stable serum creatinine (*n* = 15) (23.20 ± 22.90 vs. 16.72 ± 10.29, *P* = 0.465). Of the 32 patients with elevated serum creatinine at sample collection, 31 (96.9%) had an elevated dd-cfDNA level that was higher than the 8.93 BKPyVAN threshold as calculated in Table 3. Conversely, of the 15 patients with stable serum creatinine at the time of biopsy, 14 (93.3%) had an elevated dd-cfDNA level that was higher than the 8.93 BKPyVAN threshold, indicating that renal allograft injury occurred before serum creatinine elevating.

### Pathological Scores and dd-cfDNA

Anti-SV40-T IHC staining was negative on all kidney biopsies of probable BKPyVAN, possible BKPyVAN, and resolving BKPyVAN but was positive on biopsies of proven BKPyVAN. In the proven BKPyVAN group, including one stage A, 12 stage B1, 21 stage B2, five stage B3, and one stage C, no significant correlations were found between various kinds of histological signs and urine dd-cfDNA or urine dd-cfDNA% (Table 4).

**Table 4:** Spearman correlation analysis (*n* = 40).

	**dd-cfDNA%**		**dd-cfDNA**	
**Pathological score**	***r***	***P***	***r***	***P***
Glomerulitis	0.02	0.892	0.15	0.360
Transplant glomerulopathy	−0.27	0.096	0.06	0.705
Extent of anti-SV40-T IHC staining	0.09	0.562	0.20	0.218
Tubulitis	0.19	0.235	0.19	0.229
Interstitial inflammation	−0.07	0.651	−0.12	0.461
Total interstitial inflammation	0.14	0.391	0.09	0.597
Tubular atrophy	0.35	0.026	−0.03	0.876
Interstitial fibrosis	0.25	0.113	0.003	0.984

*dd-cfDNA, donor-derived cell-free DNA; IHC, immunohistochemical*.

### Repeated Detection of Urine dd-cfDNA

Repeated detection of urine dd-cfDNA was performed in four patients in the proven BKPyVAN group and five patients in the probable BKPyVAN group. The level of urine dd-cfDNA declined significantly in four with proved BKPyVAN (14.3 ng/ml vs. 6.5 ng/ml, *P* = 0.011) and in five patients with probable BKPyVAN (15.4 vs. 3.6 ng/ml, *P* = 0.021) after reducing immunosuppression, accompanied by obliteration of BK viremia (For a more detailed description of these patients, please refer to the Table 5).

**Table 5:** Following-up data in patients with repeated measurement of urine dd-cfDNA.

	**Interval time (months)**	**Serum creatinine (μmol/L)**		**Urine viral load (copies/mL)**		**Plasma viral load (copies/mL)**		**Urine dd-cfDNA fraction (%)**		**Urine dd-cfDNA concentration (ng/ml)**		**The extent of SV40-T (%)**	
		**Initial**	**Repeated**	**Initial**	**Repeated**	**Initial**	**Repeated**	**Initial**	**Repeated**	**Initial**	**Repeated**	**Initial**	**Repeated**
P1	11.6	223	210	2.9 × 10^8^	8.0 × 10^6^	1.0 × 10^5^	0	0.77	0.11	9.2	1.3	7	0
P2	9.2	154	146	1.9 × 10^9^	1.0 × 10^5^	3.0 × 10^3^	0	0.74	0.91	9.6	4.8	3	0
P3	6.1	179	248	1.1 × 10^7^	0	0	0	0.82	0.71	28.5	17.1	15	N
P4	5.3	277	191	2.9 × 10^7^	2.7 × 10^7^	2.0 × 10^3^	0	0.38	0.20	9.8	2.7	20	0
P5	6.9	127	95	4.9 × 10^9^	0	1.0 × 10^3^	0	0.89	0.32	13.6	1.7	0	0
P6	7.3	119	72	2.1 × 10^10^	2.3 × 10^4^	1.7 × 10^3^	0	0.93	0.84	28.9	5.5	0	N
P7	4.1	152	142	1.1 × 10^10^	1.5 × 10^7^	8.3 × 10^3^	0	0.87	0.75	14.9	3.4	0	N
P8	4.9	115	91	5.0 × 10^8^	2.8 × 10^6^	6.3 × 10^3^	0	0.93	0.90	9.2	2.3	0	N
P9	13	125	108	1.1 × 10^8^	2.3 × 10^6^	1.0 × 10^3^	0	0.84	0.82	10.2	5.1	0	N

*cfDNA, cell-free DNA; BKPyVAN, BK polyomavirus-associated nephropathy; P1~P4, patients in proven BKPyVAN group; P5~P9, patients in probable BKPyVAN group; N, no repeated biopsy*.

## Discussion

Studies have shown that measuring plasma dd-cfDNA is useful for the detection of allograft rejection/injury episodes in organ transplant recipients (3, 20). However, elevated dd-cfDNA isn't specific for rejection; elevated levels are also observed in BKPyVAN in sporadic reports (4, 9, 10). This large-scale clinical study systematically assessed the performance of urine dd-cfDNA and dd-cfDNA% for discriminating BKPyVAN in adult kidney transplant recipients with BKPyV viruria. The results show that both measures are able to discriminate proven BKPyVAN from resolving BKPyVAN and possible BKPyVAN. In addition, urine dd-cfDNA had better performance than urine dd-cfDNA%, serum creatinine, urine BKPyV DNA, or plasma BKPyV DNA for discriminating BKPyVAN.

Currently, there is no uniform consensus regarding the proportion or absolute value of urine dd-cfDNA for detecting renal allograft injury. Burnham et al. observed (21) an elevated proportion of urine dd-cfDNA in patients with BKPyVAN (mean 65.1%, *n* = 12) compared with patients with normal biopsies (no BKPyVAN, mean 51.4%, *n* = 4). Similarly, we found urine dd-cfDNA% was significantly elevated in biopsy-proven BKPyVAN (mean 71%) as compared with possible BKPyVAN (mean 56%). Detecting urine dd-cfDNA concentration can avoid the influence of fluctuation in total cfDNA (22). In addition, the vast majority of cfDNA in urine is from white blood cells (i.e., neutrophils and lymphocytes) undergoing a natural apoptosis process (22). The absolute quantification of urine dd-cfDNA can avoid the influence of these factors on the proportion of dd-cfDNA in the urine. This study also shows that urine dd-cfDNA compared with dd-cfDNA% had a better discrimination capacity for diagnosis of BKPyVAN.

Recently, the guideline about BKPyVAN in solid organ transplantation has been updated by Hirsch et al. (13). In this study, patients were grouped by the latest guideline, and seven patients who had negative anti-SV40-T immunohistochemical staining on allograft biopsies were diagnosed with probable BKPyVAN because of sustained plasma BKPyV DNA load >3 log_10_ copies/mL in two measurements within 3 weeks. The level of urine dd-cfDNA in probable BKPyVAN was higher than that in possible BKPyVAN but was similar to that in proven BKPyVAN, indicating kidney injury in probable BKPyVAN and proven BKPyVAN. Repeated detection of urine dd-cfDNA in five patients with probable BKPyVAN showed that the level of dd-cfDNA declined significantly after reducing immunosuppression in a median of 5.9 months with their allograft function improved rather than deteriorated, indicating remission of kidney injury. The probable BKPyVAN, which presented a negative biopsy, might be suffering from BKPyV infection in renal allograft but was misdiagnosed due to sample errors because BKPyV replicates locally in the medullary area, especially during the early stage (23). In addition, the urine dd-cfDNA% of the probable BKPyVAN group was even significantly higher than that of the proved BKPyVAN group. This may be due to the small sample size in the probable BKPyVAN group. Regardless, these results uniformly indicate that probable BKPyVAN has undergone renal allograft injury. Therefore, we recommend preemptive treatment for patients with BKPyV infection who have negative anti-SV40-T IHC staining on kidney biopsies but have elevated urine dd-cfDNA. To further confirm this conclusion, future studies should be performed to dynamically monitor urine dd-cfDNA before, during, and after BKPyV infection.

Our results show that there was no significant correlation between various kinds of histological signs and urine dd-cfDNA or urine dd-cfDNA%. This may be because these pathological damage indicators are morphological manifestations observed from very small tissue, which are not equivalent to the degree of kidney damage. In contrast, urine dd-cfDNA can reflect damage to the entire kidney.

This study showed that, among 15 patients with stable serum creatinine at diagnosis of BKPyVAN, 14 (93.3%) had an elevated dd-cfDNA level that was higher than the 8.93 BKPyVAN threshold. This result indicates that renal allograft injury has occurred before serum creatinine elevating, and monitoring urine dd-cfDNA can help recognize renal allograft injury earlier, thus triggering kidney biopsy and therapeutic intervention earlier. The level of urine dd-cfDNA declined significantly in four patients with proven BKPyVAN and in five patients with probable BKPyVAN. This result suggests that dd-cfDNA can be used to dynamically evaluate the therapeutic effect of BKPyVAN. This hypothesis can be verified in future large sample studies.

PCR-based BKPyV DNA load quantitation in the plasma is the preferred screening method for monitoring BKPyV reactivation at most transplant centers, including our own. Although qualitative PCR is sensitive for detecting plasma BKPyV replication, BK viremia has a relatively low positive predictive value (30–50%) for BKPyVAN (24), partially due to technical variation, including DNA extraction, primer design, and PCR cycling parameters (13, 25). With an AUC of 0.842, urine dd-cfDNA exhibited higher discrimination capacity compared with plasma BKPyV DNA load for discriminating biopsy-proved BKPyVAN from biopsy-excluded BKPyVAN in our study.

Our previous study has shown that, after kidney transplantation, the plasma dd-cfDNA level decreased rapidly, following an L-shaped curve, but the level increased markedly when an acute rejection episode occurred (15). In this study, urine dd-cfDNA levels fluctuated depending on the BKPyV infection state. The dynamic decline of urine dd-cfDNA, accompanied by obliteration of viremia, strongly suggests that kidney damage exists in probable BKPyVAN and remission of intrarenal injury after effective treatment. A similar trend was observed in proven BKPyVAN. In addition, the lower level of urine dd-cfDNA in resolving BKPyVAN was consistent with the corresponding clinicopathological state, including negative viremia, negative anti-SV40-T IHC staining on kidney biopsies, resolution of tubulointerstitial inflammation, stable serum creatinine, and persistent low copy number of urine BKPyV DNA (12, 14).

In this study, we introduced a hybrid capture sequencing approach for the quantification of dd-cfDNA based on 6200 SNPs from whole-genome. This method is not limited by the gender of the transplant recipient because the strategy of using the Y chromosome for dd-cfDNA quantification can be used only in gender-mismatched kidney transplantation (26). However, this study has some limitations. Urine dd-cfDNA was primarily assessed on a “for-cause” basis but not “for-protocol.” This may maximize the prevalence of BKPyVAN in the study cohort to a level higher than typically observed in the general kidney transplant population. The purpose of this study was to assess the value of urine dd-cfDNA for discriminating BKPyV-caused renal allograft injury in kidney transplant recipients with BKPyV viruria. Since all patients in this study had BKPyV viruria, the conclusion cannot be generalized to other patients without BKPyV infection. Moreover, there was no patient with urinary BKPyV infection combined with biopsy-proved T cell-mediated rejection (TCMR), so it was not able to provide more value for distinguishing BKPyVAN and TCMR. Our results provide clues to whether BKPyV-caused renal allograft injury has occurred in patients with BKPyV infection. Elevated urinary dd-cfDNA levels can prompt more targeted renal biopsy in patients with BKPyV infection. Declining urinary dd-cfDNA levels suggests a resolution of BKPyVAN under effective treatment.

## Conclusions

The results of this study suggest that the absolute quantification of urine dd-cfDNA could serve as an indicator for discriminating BKPyVAN injury in patients with BKPyV infection. Larger and longitudinal studies are necessary to assess the discrimination capacity of urine dd-cfDNA for serial surveillance in kidney transplant recipients.

## Data Availability Statement

The raw data supporting the conclusions of this article will be made available by the authors, without undue reservation.

## Ethics Statement

The studies involving human participants were reviewed and approved by the Ethics Committee of the First Affiliated Hospital of Sun Yat-sen University. The patients/participants provided their written informed consent to participate in this study.

## Author Contributions

GH and JQ designed the study, reviewed and edited the whole manuscript. X-TC, W-FC, and JL interpreted data and wrote the manuscript. R-HD and YH reviewed the whole manuscript. S-CY and W-FC as pathologists and evaluated the slides. P-SC performed laboratory testing. L-ZC and C-XW collected, analyzed, and interpreted data. H-TL performed cell-free DNA detection. T-YJ conducted bioinformatics analysis. All of the authors have read, approved the manuscript, and made an important contribution to the manuscript.

## Conflict of Interest

The authors declare that the research was conducted in the absence of any commercial or financial relationships that could be construed as a potential conflict of interest.

## Abbreviations

BKPyVAN: BK polyomavirus-associated nephropathy
BKPyV: BK polyomavirus
PCR: polymerase chain reaction
cfDNA: cell-free DNA
dd-cfDNA: donor-derived cell-free DNA
gDNA: genomic DNA
SNP: single-nucleotide polymorphism
IHC: immunohistochemical
ROC: receiver operating characteristic
AUC: area under the ROC curve
TCMR: T cell-mediated rejection.
